# Referral patterns for specialist child and adolescent mental health services in the Republic of Ireland during the COVID-19 pandemic compared with 2019 and 2018

**DOI:** 10.1192/bjo.2021.48

**Published:** 2021-05-03

**Authors:** Fiona McNicholas, Ian Kelleher, Elma Hedderman, Fionnuala Lynch, Elaine Healy, Therese Thornton, Edwina Barry, Lisa Kelly, James McDonald, Keith Holmes, Glenda Kavanagh, Maria Migone

**Affiliations:** Department of Child & Adolescent Psychiatry, School of Medicine and Medical Science, University College Dublin, Ireland; Children Health Ireland, Dublin, Ireland; and Lucena Clinics, St. John of God Community Services, Ireland; Lucena Clinics, St. John of God Community Services, Ireland; Lucena Clinics, St. John of God Community Services, Ireland; Lucena Clinics, St. John of God Community Services, Ireland; Lucena Clinics, St. John of God Community Services, Ireland; Lucena Clinics, St. John of God Community Services, Ireland; Lucena Clinics, St. John of God Community Services, Ireland; Lucena Clinics, St. John of God Community Services, Ireland; Lucena Clinics, St. John of God Community Services, Ireland; Lucena Clinics, St. John of God Community Services, Ireland; Lucena Clinics, St. John of God Community Services, Ireland; Lucena Clinics, St. John of God Community Services, Ireland; Department Academic Child & Adolescent Psychiatry UCD, Dublin

**Keywords:** CAMHS, COVID-19, referrals rates, activity, mental health

## Abstract

**Background:**

Countries worldwide are experiencing a third wave of the coronavirus disease 2019 (COVID-19) pandemic. Government-imposed restrictive measures continue with undetermined effects on physical and mental health.

**Aims:**

To compare child and adolescent mental health services (CAMHS) referrals over 11 months (January–November) in 2020, 2019 and 2018 and examine any impact the different phases of the COVID-19 restrictions might have on referral rates.

**Method:**

Monthly CAMHS Health Service Executive data were examined, covering a catchment population of 260 560 or 12.7% of all youth (age group 0–18 years) in Ireland. The total number of urgent and routine referrals, appointments offered, rates of non-attendances and discharge outcome are presented.

**Results:**

There was a significant drop in referrals in 2020, compared with prior years (χ^2^ = 10.3, d.f. = 2, *P* = 0.006). Referrals in 2020 dropped from March to May by 11% and from June to August by 10.3%. From September, both routine and urgent referrals increased by 50% compared with previous years (2018/2019), with the highest increase in November 2020 (180%). Clinic activity also increased from September, with double the number of out-patient appointments offered, compared with previous years (*χ*^2^ = 5171.72, d.f. = 3, *P* < 0.001) and lower (6.6%) rates of non-attendance (*χ*^2^ = 868.35, d.f. = 3, *P* < 0.001).

**Conclusions:**

In 2020, following an initial decline, referrals to CAMHS increased consistently from September. Such unprecedented increase in referrals places further strain on services that are already underresourced and underfunded, with the likelihood of increased waiting lists post COVID-19. It is envisaged that once the pandemic is over, resources will be even more constrained, and CAMHS will be urgently in need of additional ring-fenced funding.

## Background

As with many other countries, the Republic of Ireland (ROI) is entering another year of living with the coronavirus disease 2019 (COVID-19) pandemic and associated restrictions. Unprecedented restrictions were initially imposed on 12 March 2020, following the declaration by the World Health Organization that the coronavirus infection had reached the status of a global pandemic. Schools, universities and all non-essential businesses were closed; ‘stay-at-home’ orders and severe restrictions on social gatherings and movements were imposed.

The direct impact for the 2 049 489 youth population in the ROI was of acute disruption to their lives, changes in their education, with cancellation of exams and a shift to online learning, restricted physical and social distancing and cessation of sporting and training events. For some adverse financial and health costs to family members and inability to visit, or at times appropriately grieve for, grandparents were also experienced as negative and anxiety provoking. The associated high morbidity and mortality of COVID-19 led to fear that the healthcare system would become overwhelmed and the initial 3-month restrictions were adhered to despite lack of understanding as to the exact toll or unintended consequences on children, families and society.

Subsequent restrictions were re-applied following the summer months, although schools remained open. Currently, many countries have once again instigated varied restrictions, with a return to closure of non-essential businesses, hospitality, sporting events, school and colleges, with stay-at-home orders in place in the ROI. Publicly aired voices of discontent and accusations levelled at youth for excessive and reckless socialising has added a layer of guilt and a sense of lack of appreciation of their lot. The gravity and complexity of these unintended psychosocial consequences have yet to be measured.

## Impact on mental health

It is recognised from previous experiences that the mental health impact of a pandemic is far greater and longer lasting than any medical sequela.^1^ Evidence has been accumulating as to the negative impact of COVID-19 on the public, healthcare workers and those with pre-existing mental illness.^2^ Children are less likely than adults to be infected or have adverse medical outcomes, and although some data is emerging attesting to higher rates of anxiety, depression and post-traumatic stress in children,^3^ the extent of adverse mental health outcomes has yet to be robustly studied.^4^ Some data from voluntary organisations and helplines have reported increased contact, suggesting an increase in psychological distress.^5^ Higher reports of domestic violence have also been reported.^6^ However, rates of actual increase in mental illness or referral to child and adolescent mental health services (CAMHS) within the ROI have so far not been available.

## Aims

This study aims to:

- report on all referrals from January to November in 2020 received by five CAMHS in Dublin. Between them these clinics are responsible for a catchment area of 260 560 youth or 12.7% of youth in the ROI;
- compare referral rates and management of referrals in 2020 with a similar time period (January–November) in 2018 and 2019;
- examine any differential response based on phases of COVID-19 restrictions in 2020. The phases examined are phase 0 (pre-COVID): January–February; phase 1, March–May; phase 2: June–August and phase 3: September–November.

## Method

Lucena clinic, part of the St John of God Community Mental Health Services, has a contractual agreement with the Irish Health Service Executive (HSE) to provide specialist CAMHS to youth residing in the community healthcare organisation (CHO) area 6 and part of CHO area 7. This service is consultant led and delivered by ten consultants across five multidisciplinary teams. The youth population is 116 264 in CHO area 6 and 144 296 in CHO area 7, totalling 260 560 youth or 12.7% of all youth in the ROI.

Monthly out-patient activity data is provided annually to the HSE detailing the total number of urgent and routine referrals, the speed of clinic response, the number of monthly appointments offered, the number of patients that cancelled or did not attend and the number of patients discharged.

These anonymous and publicly available data were used to compare activity pre-COVID-19 and during COVID-19. Overall CAMHS activity was compared for 2018, 2019 and 2020. The following time frames were examined (Appendix): phase 0: pre-COVID-19 from 1 January to 29 February 2020; phase 1: 1 March to 31 May, represented the first phase of lockdown, during which schools, colleges and all non-essential businesses were closed; phase 2: 1 June to 31 August was linked with the easing of restrictions; and phase 3: 1 September to 30 November 2020, aligned with further periods of restriction but with schools open.

Out-patient activity was examined with reference to each of the above time frames and compared with a similar time period in 2018 and 2019. Data is provided both for the full time period and for each month to control for difference in duration across the time periods assessed. Chi-squared (goodness of fit) analysis was conducted to examine if there is a statistically significant difference between groups, with standard residuals, the difference between the actual and observed cell counts, helping identify the source.^7^ Values >|2| show significantly greater deviation than expected, with the plus (+) and minus (−) sign showing the direction of the diversion (+, more; −, less).

This study was performed in accordance with the Declaration of Helsinki. Ethics approval was not required as only administrative data is reported on.

## Results

Data on referrals were available for a 3-year period (2018–2020). Referrals to CAMHS from January–November in 2020 were lower than previous years (*χ*^2^ = 10.3, d.f. = 2, *P* = 0.006) (Table 1). Compared with a similar 11-month time period in 2018 and 2019, they dropped by 8.9% and 6.6%, respectively.

**Table 1:** January–November referrals and discharges in 2018, 2019 and 2020

	2018	2019	2020	χ^2^ test (d.f.)^a^	*P*
Referrals					
*n*	2361	2305	2152	10.3 (2)	0.006
Standard residual	+2.27	+0.83	−3.10	–	–
Referrals accepted					
*n* (%)	1943 (82.3)	1847 (80.1)	1517 (70.5)	56.45 (2)	<0.001
Standard residual	+4.13	+1.85	−5.99	–	–
Of those accepted *n* (%) seen within 13 weeks					
*n* (%)	525 (27.0)	956 (51.8)	716 (47.2)	127.37 (2)	<0.001
Standard residual	−9.38	+10.12	−0.74	–	–
Urgent referrals received, *n* (%)	NA	100 (4.3)	89 (4.1)	0.64 (1)	0.424 (NS)
Urgent referrals seen within 3 days, *n* (%)	NA	94 (94)	65 (73)	5.28 (1)	0.021
Total discharges					
*n*	2380	2400	2105	23.68 (2)	<0.001
Standard residual	+2.17	+2.68	−4.86	–	–
Discharge to general practitioner					
*n* (%)	2222 (93.4)	2249 (93.7)	1870 (88.8)	42.31 (2)	<0.001
Standard residual	+2.89	+3.61	−6.49	–	–
Discharge to other CAMHS (in-patient/hospital-based paediatric liaison psychiatry)					
*n* (%)	93 (3.9)	82 (3.4)	157 (7.3)	29.65 (2)	<0.001
Standard residual	−2.06	−3.34	+5.93	–	–
Discharges to AMHS					
*n* (%)	43 (1.8)	38 (1.6)	29 (1.4)	2.75 (2)	0.253
Standard residual	+1.28	+0.27	−1.55	–	–
Discharge to community-based primary care counselling services					
*n* (%)	22 (.9)	31 (1.3)	49 (2.3)	11.12 (2)	0.004
Standard residual	−2.52	−0.63	+3.15	–	–

NS, not significant; NA, not available; CAMHS, child and adolescent mental health services; AMHS, adult mental health services.

a. χ^2^ among columns.

Significantly fewer referrals (70.5%) were accepted (*χ*^2^ = 56.45, d.f. = 2, *P* < 0.001) in 2020. A total of 47.2% of referrals received in 2020 were seen within 13 weeks (Table 2). Significantly fewer patients were discharged in 2020 (Table 1) (*χ*^2^ = 23.68, d.f. = 2, *P* < 0.001). Additionally fewer patients were discharged back to their general practitioner alone (88.8% in 2020 compared with over 93% in prior years) (*χ*^2^ = 42.31, d.f. = 2, *P* < 0.001). There was a significant increase in onward referrals (*n* = 157) to other CAMHS (in-patient or liaison services) suggesting increased complexity compared with 2019 (*n* = 82) or 2018 (*n* = 93) (*χ*^2^ = 29.65, d.f. = 2, *P* < 0.001).

**Table 2:** Child and adolescent mental health services out-patient department (OPD) activity by coronavirus disease 2019 (COVID-19) phases of lockdown and comparison with 2019

All clinics	January–February, phase 0	March–May, phase 1	June–Aug, phase 2	September– November, phase 3	*χ*^2^ (d.f.) *P*, among columns
OPD offered, 2020					
*n* (*n* per month)^a^	9041 (4520)	12 913 (4304)	11 852 (3950)	20 363 (6788)	5171.72 (3) <0.001
Standard residual	−44.66	−6.24	−16.77	+67.68	–
OPD offered, 2019					
*n* (*n* per month)^a^	10 130 (5065)	12 444 (4148)	11 643 (3881)	15 004 (5001)	1013.62 (3) <0.001
Standard residual	−22.64	+1.44	−6.89	+28.09	–
OPD offered, *χ*^2^ (d.f.) *P*, comparing 2020 and 2019	61.86 (1) <0.001	8.6746 (1) 0.003	1.8592 (1) 0.175	812.02 (1) <0.001	–
DNA, 2020					
*n* (*n* per month)^a^	745 (373)	604 (201)	1059 (353)	1351 (450)	355.42 (3) <0.001
Standard residual	−7.34	−12.65	+4.49	+15.49	–
% DNA of those offered, 2020	8.2	4.7	8.9	6.6	–
Cancelled,^b^ 2020					
*n* (*n* per month)^a^	1516 (758)	3605 (1202)	1194 (398)	1723 (574)	1759.78 (3) <0.001
Standard residual	−12.71	41.10	−21.01	−7.38	–
% cancelled of those offered, 2020	15.0	28.0	10.1	8.5	–
Total DNA or cancelled, 2020					
*n*	2261	4209	2253	3074	868.35 (3) <0.001
Standard residual	−14.63	+26.79	−14.80	+2.65	–
% DNA or cancelled of those offered, 2020	25.0	32.6	19.0	15.1	–

SR, Standard residual; DNA, did not attend – no advanced notice given.

a. Monthly averages are in brackets.

b. Cancelled: clinic advised ahead of time about cancellation either by clinician or family.

Information on urgent referrals was available for 2019 and 2020 only. There was no difference in proportion of urgent referrals in 2020 (4.1%) or 2019 (4.3%) (*χ*^2^ = 0.64, d.f. = 1, *P* = 0.424, not significant). However, significantly fewer were seen within 3 working days in 2020 (73%) compared with 2019 (94%), suggesting capacity overload (*χ*^2^ = 5.28, d.f. = 1, *P* = 0.021) (Table 1).

### Referrals with reference to COVID-19 phases of restrictions and comparison with 2019 and 2018

Although total referrals were lower in 2020 than previous years, subsequent analysis was conducted to examine the impact of COVID-19 on referral rates according to the pandemic timeline and level of imposed restrictions.

**Fig. 1:**
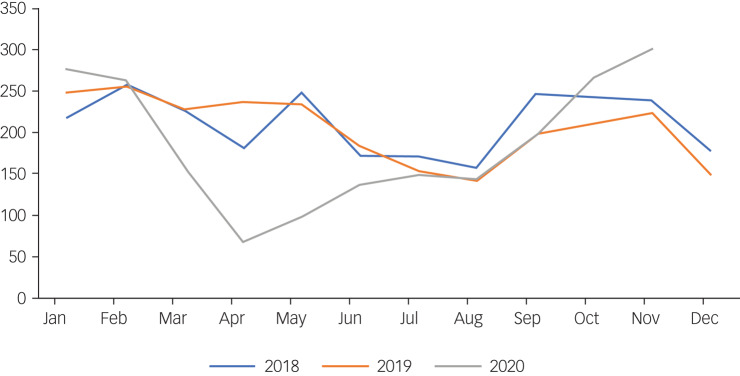
Monthly trend in referrals rates by month for 2018 to 2020.

In previous years, 2018/2019, rates of referrals to CAMHS typically dropped during holiday times, with lows at Easter (April), and summer (June–August), picking up again from September, and peaking in October (Fig. 1). This pattern did not hold during 2020.

#### Phase 0 (1 January–29 February)

The total CAMHS referrals for 2020 were higher (*n* = 538) compared with 2019 (*n* = 501) and 2018 (*n* = 475). However, this was not significant (*χ*^2^ = 3.972, d.f. = 2, *P* = 0.137).

#### Phase 1 (1 March–31 May)

The total number of referrals to CAMHS for March–May 2020 were lower (*n* = 324) compared with 2019 (*n* = 695) and 2018 (*n* = 655), highlighting a significant drop in 2020 at the onset of COVID-19 (*χ*^2^ = 148.627, d.f. = 2, *P* < 0.001).

#### Phase 2 (1 June–31 August)

The total number of referrals in the summer months 2020 (*n* = 428) were lower compared with 2019 (*n* = 477, 10.3% lower) and 2018 (*n* = 501, 14.6% lower). Although no overall significant differences existed among the years 2019 and 2020 (*χ*^2^ = 5.908, d.f. = 2, *P* = 0.052), referrals in 2020 were significantly lower than 2018 (*χ*^2^ = 5.736, d.f. = 1, *P* = 0.017).

#### Phase 3 (1 September–30 November)

The total number of referrals to CAMHS in phase 3, 2020 increased significantly (*n* = 762) and was higher than previous years (*n* = 632 in 2019 and *n* = 730 in 2018) (*χ*^2^ = 12.96, d.f. = 2, *P* = 0.002). On examination of the numbers of urgent referrals in this period, there was a significant increase of 180%, (*n* = 42) compared with 2019 (*n* = 15; *χ*^2^ = 12.789, d.f. = 1, *P* < 0.001)

### CAMHS out-patient department activity in 2019 and 2020

Despite fewer referrals in 2020, out-patient activity was 10.1% higher with most of the increased activity in September–November (*χ*^2^ = 5171.72, d.f. = 3, *P* < 0.001) (Table 2). Fewer patients were discharged in 2020 than expected (Table 2) (*χ*^2^ = 23.68, d.f. = 2, *P* < 0.001).

#### Phase 0 (1 January–29 February)

Fewer out-patient appointments (4520 per month) were offered in 2020 during this period compared with 2019 (*n* = 5065; Table 2). Also, 25% of all appointments offered were either cancelled (16.8%) by the clinician or family, or the family failed to attend without explanation (8.2%).

#### Phase 1 (1 March–31 May)

During the first lockdown period in 2020, out-patient activity reduced (*n* = 4304/month) compared with clinic activity in phase 0 of 2020 (*n* = 4520/month). However, activity rates were still higher than monthly averages in 2019 (*n* = 4148/month). Rates of non-attendances for any reason were very high during this period (*n* = 4209, 32.6%), with most of these being cancelled ahead of time (*n* = 3605, 28%).

#### Phase 2 (1 June–31 August)

In total, *n* **=** 3950 appointments were offered on average each month over the summer months, with rates similar to 2019 levels (*n* = 3881/month). Rates of non-attendances for any reason reduced during this period (*n* = 2253, 19%).

#### Phase 3 (1 September–30 November)

Out-patient activity was significantly raised during this phase with monthly averages of *n* = 6788 appointments offered, almost double the rate of the summer period (*χ*^2^ = 5171.72, d.f. = 3, *P* < 0.001), and 50% higher than January–February levels. They were also 35.7% higher than the similar time period in 2019 (*n* = 5001/month). Proportionally more of these appointments were seen, as rates of non-attendances for any reason was low (*n* = 3074; 15.1%; *χ*^2^ = 868.35, d.f. = 3, *P* < 0.001). (Table 2)

## Discussion

### Referrals

Overall, referrals to CAMHS fell during 2020 and were 11% lower than 2019 and 4.2% lower than 2018. This difference was most evident in phase 1 (1 March–31 May), which signalled the closure of non-essential businesses, schools and colleges. A stay-at-home order was put in place, exercise was limited to 5 km from home and social gatherings forbidden. Referrals in this period dropped by 53% compared with 2019 and 51% compared with 2018 (Figure 1). Referral rates during the summer months 2020 (1 June–31 August), when restrictions had been lifted, remained lower than previous years. There were 10% fewer referrals received during phase 2 compared with 2019, and 15% fewer than 2018.

This drop in presentation to mental health services immediately post restrictions has also been reported in community mental health^8^ and hospital settings.^9^ Tromans and colleagues, examining UK mental health referral data, in the 8-week period before (27 January–22 March 2020) and after (23 March–17 May 2020) the first UK lockdown, reported a significant reduction in referrals to all services (37%), with rates in CAMHS falling by 51%.^8^ Examining psychiatry referrals to the emergency department during the initial 8-week COVID-19 restrictions in Ireland (16 March 2020 to 10 May 2020), McAndrew and colleagues also reported a significant reduction in weekly referrals by 21%, compared with previous years.^9^ In studies where the time period was extended, a rebound in attendances has been noted. Presentations with self-harm dropped by 35% from March to April but rose by 104% for adults from April to May.^10^ Rates of children attending emergency departments for either medical or mental health presentations also fell during this initial 3-month period (March–May).^11^

### Reasons for reduced referrals during phase 1 and phase 2 (March–August 2020)

There are a number of possible explanations for reduced referrals to CAMHS in early 2020. For one, it could reflect a reluctance of the patient or the family to seek help during lockdown, including low attendance at general practitioner services, the gate-keepers to CAMHS referral. Research in the UK suggests that nearly 1 in 12 parents (8.3%) of children with a ‘probable mental disorder’ decided not to seek help and more than 1 in 5 of 17- to 22-year-old young people themselves with a probable mental disorder chose not to seek help.^11^ It could also reflect difficulty accessing general practitioner services because of prioritisation of COVID-19-related activity. It is also possible that reduced referrals could reflect a genuine reduction in mental health problems, arising, for example as a result of school closures.

Schools represent a major source of stress for many young people, including from academic, social and behavioural perspectives. What is more, for many young people, parents may have been more available to them during this period given workplace closures and home working. In the Mental Health of Children and Young People in England study, a significant minority of youth reported that lockdown had made their life better.^11^

Although these factors might account for the initial decrease, they do not persist. Research indicates that as COVID-19 progressed, presentations of youth to emergency departments increased.^12^ Leeb and colleagues reported that whereas mental health-related emergency department attendances decreased sharply from mid-March through early April, they increased steadily through to October 2020.^12^

Attendances at non-specialist services also increased. For example, domestic violence helplines saw an increase in calls,^2^ the Irish youth mental health charity Jigsaw reported a 400% increase in traffic to its e-mental health platform^5^ and Bodywhys, the eating disorder organisation, reported a 110% increase in online supportive engagement.^13^

A series of surveys conducted by Young Minds UK, reported that youth with prior mental health difficulties (*n* > 2000) had decreasing levels of mental health well-being as the pandemic progressed and experienced increased difficulty accessing services.^14^ They found that 32% of respondents believed that COVID-19 had made their mental health ‘much worse’ in March 2020, increasing to 41% by June 2020. Feelings of loneliness and isolation were reported by 87%, despite the majority (71%) staying in touch with friends. A total of 68% of students reported that returning to school in September 2020 after a period of absence lead to deterioration in their mental health, with 40% reporting there was no school counsellor available, and 23% experiencing that mental health school supports were fewer than pre-COVID-19.^14^ Immediately post-lockdown, 31% of youth surveyed reported being no longer able to access previously attended mental health services despite their ongoing need.^14^ Follow-up data from England's Mental Health of Children and Young People survey suggest an increase in rates of ‘probable mental health conditions’ from 10.8% in 2017 to 16% in July 2020.^15^ The ongoing and increasing need for CAMHS services as COVID-19 progresses has been evident in this study by the large increase in referrals in phase 3.

### Clinical complexity

A higher proportion of patients referred to CAMHS during COVID-19 were deemed urgent, and increased numbers were in need of onward referral from CAMHS to other specialist services, which included in-patient and hospital-based paediatric liaison psychiatry services. In 2020, 7.5% of all patients seen were transferred on to other CAMHS, which is double the rate seen in 2019 (3.4%) within the Lucena service and higher than prior national averages (4.5%).^16^ Although data is not publicly available on the clinical profile of referrals, anecdotal reports by clinicians indicate an increase in referral complexity, with a higher proportion with suicidal ideation and with eating disorders.

Internal referrals to the eating disorder family-based treatment team within Lucena saw a jump from 19 referrals in 2019 to 42 in 2020.^17^ This increase in maladaptive and disordered eating has also been reported in the UK.^18^ Among youth in the UK, new referrals have increased by 46% during the pandemic^19^ and there has been a 20% increase in emergency admissions to acute hospitals among adults with eating disorders.^20^ Increased clinical complexity has been highlighted in studies examining presentations to paediatric emergency departments.^21^

Data collected from March to May 2020 from 23 emergency departments internationally, corresponding with phase 1 of this study, showed that despite a decrease in overall referrals, clinical complexity increased and the proportion of youth presenting with self-harm increased.^21^ The increased awareness of rising rates of self-harm among youth was already a cause for concern pre- COVID-19 in the UK^22^ and the ROI,^23^ but had been in parallel with improvements in evidence-based treatments.^24^ Increased presentation and lethality of self-harm among adults during COVID-19 has also been reported.^11^

### Activity

Relative to January–February 2020, clinic activity declined during the immediate lockdown period (March–May) and over the summer months. However, appointments increased sharply from September to November, with a 71.8% increase in appointments offered per month with respect to the summer months, and 35.6% higher than the equivalent period in 2019. Non-attendance rates were also higher during the earlier COVID-19 period such that 32.6% of all patients from phase 1 did not attend, 19% did not attend from phase 2, and 25% did not attend from phase 0. By phase 3 (1 September to 30 November) the overwhelming majority of appointments offered were attended (84.9%) and the non-attendance rate without prior cancellation was 6.6%, which is below previous national averages (8.5%).^25^ Taking both advanced cancellations (*n* = 1723) and ‘no-shows’ or did not attend (*n* = 1351), overall non-attendance during this period was 15.1%.

### Capacity

The ongoing demand on already overstretched services is a cause for concern. Fewer patients who were deemed in urgent need of an appointment were able to be seen within a 3-day period, in 2020 compared with 2019, suggesting capacity overload in 2020. This poses a risk of not only leading to prolonged stress in families, but also contributing to moral injury among clinicians, unable to provide a necessary and timely service, and exposing them to additional risk of burnout. It has long been recognised that CAMHS in the ROI are underfunded and underresourced, but as yet urgent and targeted investment has not occurred. Prior to the COVID-19 pandemic, CAMHS were already experiencing an increased volume of referrals, with the HSE reporting a 24% increase in accepted referrals between 2012 and 2018.^25^ In that same period, national staffing levels were just 58% of the clinical staffing levels recommended in the Irish mental health policy document, A Vision for Change.^16^ Given that 25% of the ROI population is under 18 years of age this chronic underresourcing continues to be a public health crisis. To add the recognised increased demand, consequent to COVID-19, on an already depleted service provides the perfect storm for staff burnout. High rates of staff burnout have already been reported by consultants working in CAMHS.^26^ The increased referral rates to CAMHS, increased activity and recognised adverse psychological impact of COVID-19 on clinicians expose a vulnerable service.

### Limitations

The referral data presented is from a group of five CAMHS from one organisation (St John of God Lucena Service) and although it covers a large youth catchment area, it may not be generalisable to other regions in Ireland. Furthermore, as an urban group, it may not accurately reflect rural services with reduced access to community-based mental health supports or paediatric emergency departments.

Data available is limited to referral numbers and out-patient activity level, with no supporting clinical or demographic information to allow an examination of any differential increases imposed by COVID-19 or the restrictions by clinical type or sociodemographic factors. The available reporting data to the HSE is based on monthly returns, and allowed the authors present data over the timeframes outlined (Appendix). The time periods are close approximations to the level of COVID-19 restrictions imposed, rather than an exact fit, as restrictions introduced in Ireland did not commence on the first day of each month. However, they closely align with periods in which schools were closed as part of COVID-19 restrictions (phase 1), schools were on vacation (phase 2) or schools were open despite other COVID-19 restrictions (phase 3). The time periods chosen also mirror those chosen by other Irish studies.^8,9^

### Implications

Data presented in this study show an initial 6-month decline in CAMHS referrals in 2020 followed by a sharp increase from September, peaking in November. The immediate decline in referrals post-lockdown might erroneously have suggested a reduced need for mental health youth services. However, subsequent months saw increased referrals and an increased demand for CAMHS.

The lower rate of infection, lower morbidity and mortality in youth from COVID-19 has not been met with modified restrictions, and concerns have correctly been raised about the ‘wider collateral damage to children’ of restrictions on education, socialisation, development and psychological well-being.^27^ The adverse impact on clinicians within CAMHS is also a concern. Careful planning, including data-collection systems that allow more in-depth examination of clinical information, and resourcing of CAMHS is urgently needed in the ROI to cope with the expected ongoing demand for services.

## Data Availability

The data that support the findings of this study are available from the corresponding author, F.M., upon reasonable request.

## Appendix

### Phases of coronavirus disease 2019 (COVID-19) restriction and data collection periods

**Table:**

	Study dates	Main restrictions, Republic of Ireland (ROI)	Main restrictions, UK
Phase 0: pre-COVID-19	1 January–29 February	No restrictions in place	No restrictions
Phase 1	1 March–31 May	From 12 March, closure of schools, colleges and child care facilities. Limited outdoor gatherings and travel restricted to within 2 km radius. Hospital visits restricted. Pubs and restaurants closed	A national lockdown 23 March with closure of schools, colleges, non-essential shops, gradually loosening by middle to end May
Phase 2	1 June–31 August	Indoor gatherings of 50 people, outdoor up to 200. Summer camps open, sports and limited travel allowed. Restaurants and pubs open with some restrictions	UK go into phase 2, with re-opening of shops, schools, hospitality and outdoor leisure activities
Phase 3	1 September–30 November	Stay-at-home order re-enforced, essential retail only open, pubs and restaurants closed. No social gatherings and limitations on funerals and weddings. Exercise within 5 km of home. Schools and colleges open	Restriction gradually reintroduced from September on. Ban on indoor gatherings, curfew imposed, schools, eateries, pubs closed, with fines for breach of rules
